# Life Beyond 65: Changing Spatial Patterns of Survival at Older Ages in the United States, 2000–2016

**DOI:** 10.1093/geronb/gbz160

**Published:** 2020-01-29

**Authors:** Yana C Vierboom, Samuel H Preston

**Affiliations:** 1 Population Health Lab, Max Planck Institute for Demographic Research, Rostock, Germany; 2 Department of Sociology and Population Studies Center, University of Pennsylvania, Philadelphia

**Keywords:** Demography, Geography, Health disparities, Life expectancy, Mortality

## Abstract

**Objectives:**

To identify levels and trends in life expectancy at age 65 (e_65_) by geographic region and metropolitan status in the United States.

**Methods:**

Using county-level data on population and deaths from the Census and National Center for Health Statistics, we consider spatial inequality in e_65_ across 4 metropolitan types and 10 geographic regions from 2000 to 2016. We examine whether changes in e_65_ are driven by mortality developments in metro types or geographic regions, and compare spatial patterns in the United States to mortality trends in other Organization of Economic Cooperation and Development (OECD) countries. We use decomposition and regression methods to estimate the contributions of 10 causes of death to changes and inequalities in e_65_.

**Results:**

Life expectancy at age 65 increased in all spatial units from 2000 to 2016. Areas with higher e_65_ in 2000 also experienced larger gains. Longevity increases were greatest in large metropolitan areas and coastal regions. Nonmetropolitan areas and the interior lagged far behind not only other parts of the United States but all OECD comparison countries. Metropolitan status was a better predictor of mortality changes than geographic region. Circulatory diseases and diseases associated with smoking were the principal sources of life expectancy gains and spatial differentiation in those gains. Larger gains in smoking-related mortality accounted for greater improvements among men than women.

**Discussion:**

Even at advanced ages, large geographic disparities in life expectancy remain. And as mortality has declined, these disparities have widened. Public health efforts should pay special attention to identifying and ameliorating the sources of lagging life expectancy in nonmetropolitan regions.

As death rates in the United States have declined and life expectancy at birth has advanced, more people than ever in the nation’s history are living to older ages. In the official life tables of 2016, 84% of newborns will survive to age 65 (Arias & Xu, 2018), compared to 71% a half century earlier (National Center for Health Statistics, 1968). Longer lives have major implications for family life cycles (Agree, 2019), patterns of work life (Loichinger & Weber, 2016), and durations of disability (Crimmins, 2015). There are also major fiscal consequences. Mortality declines above age 62 present an unalloyed fiscal disadvantage for the Social Security Administration’s Trust Fund Balance by increasing the number of beneficiaries faster than payroll (Trustees, 2019). Because the vast majority of deaths occur above age 65, patterns in later-life mortality are also increasingly consequential for shaping inequalities in life expectancy at birth. A growing fraction of the variance in life expectancy at birth from population to population is attributable to variation in death rates above age 65 (Elo, Hendi, Ho, Vierboom, & Preston, 2019; Klenk, Keil, Jaensch, Christiansen, & Nagel, 2016).

Despite the far-reaching implications of changing mortality levels at older ages, there are virtually no studies of the changing patterns of mortality at older ages in the United States and the possible factors responsible. Instead, most of the recent attention to variation in mortality in the United States has been directed towards the working ages, where trends have been problematic (Case & Deaton, 2015, 2017; Elo et al., 2019; Shiels et al., 2017; Stein, Gennuso, Ugboaja, & Remington, 2017; Woolf et al., 2018). Rising mortality in the working ages reduced life expectancy at birth between 2015 and 2017 (Arias and Xu, 2018; Murphy, Xu, Kochanek, & Arias, 2018), primarily as a result of increases in drug poisoning at these ages (Dowell et al., 2017). In contrast, changes in mortality from drug overdose are a minor factor at ages above 65 (Elo et al., 2019), where mortality is dominated by chronic disease processes such as cardiovascular disease, cancer, chronic lower respiratory diseases, dementia, and diabetes. Because of the heavy concentration of chronic disease mortality at ages above 65, these ages warrant separate attention.

In this paper, we examine spatial levels and trends in sex-specific mortality above age 65 from 2000 to 2016. Our focus is on differences in patterns of change by metropolitan status and geographic region, treating the two spatial classifications both separately and together. We situate patterns in a broader context by extending some comparisons to 1990 and by introducing international data. We also identify the causes of death driving changes in mortality across regions and metropolitan types.

## Method

### Data and Spatial Classification

We use age-, sex-, and county-specific data on annual deaths and underlying cause of death from Multiple Cause of Death files provided by the National Center for Health Statistics (NCHS) for years 1990–2016. We estimate person-years of exposure using vintage 2016 NCHS/Census bridged-race population estimates by age, sex, and county (postcensal for 2011 forward and intercensal for earlier years).

We consider trends in 40 spatial units representing 10 geographic regions and 4 types of metropolitan status (Supplementary Appendix Table 1). Geographic regions include the nine Census divisions and Appalachia, as defined by the Appalachian Regional Commission. Appalachian counties include all of West Virginia and counties from 12 other states and are excluded from their overlapping Census regions. We determine a county’s metro status in 2013 using the Economic Research Service (ERS) classification, modified by NCHS (Ingram & Franco, 2014). Metro status consists of the following four categories: large metropolitan areas, their suburbs, medium and small metropolitan areas, and nonmetropolitan areas (details in Supplementary Appendix Table 1). We use region and metro categories, rather than states or counties, because states would obscure rural/urban differences and considering 3,000+ counties would produce excessive detail. For consistency over time, we use counties’ metropolitan classification as of 2013.

### Measures and Methods

Our principal measure of mortality is life expectancy at age 65 (e_65_), which is calculated using standard methods (Preston, Guillot, & Heuveline, 2001) and takes account of the growth rate of the population at ages 85+ (Horiuchi & Coale, 1982). To examine recent patterns of change in e_65_, we plot a time series of annual observations since 1990. Although our study primarily addresses changes in the 21st century, we extend the time series back to 1990 to provide historical context. We also employ a scatter diagram and ordinary least squares regression to examine the relation between e_65_ in 2000 and 2016.

We examine whether over-time changes in e_65_ among the 40 units are primarily associated with metropolitan status or region by comparing the variance explained by metro status or region in bivariate linear regressions of changes in e_65_ on either region or metro status. We also estimate a multivariate linear regression of mortality change in each of the 40 units on both metro status and region and use the parameters of this regression to estimate the mean predicted change in a particular category using the *margins* package in Stata. For example, to predict change in the Mid-Atlantic region, we use the regression parameters to predict change for the Mid-Atlantic region in each of the four metropolitan categories and then average those four predicted values. Repeating this process for each region removes the influence of differing distributions of metro statuses on the distribution of regional changes. The process yields an estimate of the effect of region on mortality, controlling for metro effects (and vice versa).

We consider the role of 10 mutually exclusive and exhaustive cause-of-death categories in driving the patterns observed. They include:

- Alzheimer’s disease;
- Breast, prostate, cervical, and colorectal cancers (“screenable cancers”):
- Circulatory diseases;
- Diabetes;
- Disorders of the mental or nervous systems (excl. Alzheimer’s and substance abuse disorders);
- External causes (incl. substance abuse disorders);
- Influenza and pneumonia;
- Lung cancer and respiratory diseases (“smoking-related causes of death”);
- Other cancers; and
- All other causes

The ICD-10 codes for these causes are listed in Supplementary Appendix Table 2. The list above includes two categories which are combinations of several causes. We aggregate screenable cancers—breast, prostate, colorectal, and cervical—to create an indicator of access to and quality of health services. Many deaths from these cancers are preventable by timely diagnosis and proper treatment (Ginsberg, Edejer, Lauer, & Sepulveda, 2009; Ginsberg, Lim, Lauer, Johns, & Sepulveda, 2010; Heijnsdijk et al., 2015; Plevritis et al., 2018). We also combine lung cancer and respiratory diseases to serve as an indicator of the mortality effects of smoking. A review of estimates of smoking-attributable deaths in the United States concludes that, depending on age and method, 85%–90% of lung cancer deaths among males, and 73%–87% among women, are attributable to smoking (Oza, Thun, Henley, Lopez, & Ezzati, 2011). For chronic obstructive pulmonary disease, the principal category within respiratory diseases, 80%–88% of deaths are attributable to smoking for men and 73%–87% for women (*ibid.*)

We assess the causes of death driving changes in mortality at older ages using a standard decomposition formula developed by Arriaga (1984). We perform separate decompositions for each of the 4 metro and 10 region types. To identify the causes of death responsible for changing mortality differentials across the 10 regions, we linearly regress each region’s cause-specific contribution on its change in e_65_, separately for each cause of death. Given the additivity of causes, the slope of each cause-specific regression indicates the proportion of the variation in all-cause mortality change across spatial units that is attributable to that cause of death (Preston, 1976). Added across causes, the slopes sum to 1.00.

All analyses were performed using Stata version 16 (StataCorp).

## Results

### Changes in Life Expectancy at Age 65

**Figure 1.**
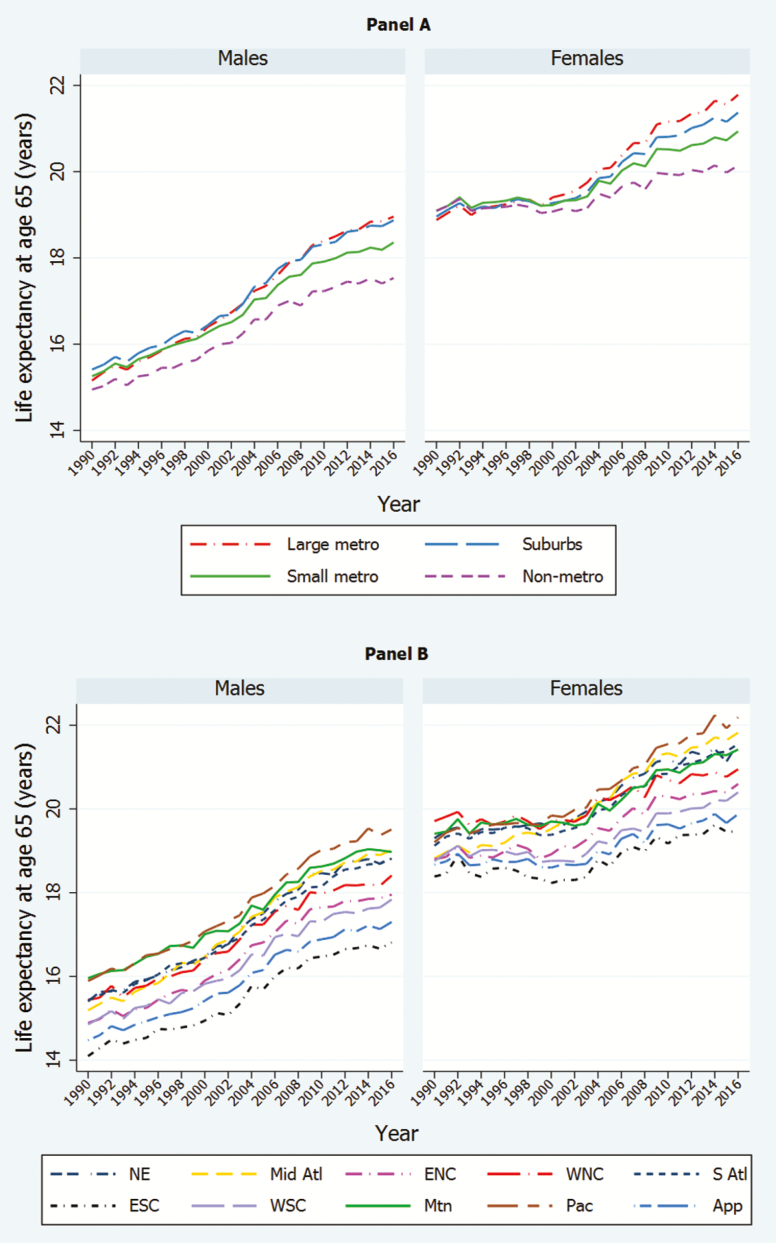
Life expectancy at age 65 by metropolitan status (Panel A) and region (Panel B), 1990–2016. *Note*: Source: Multiple cause of death files from the National Center for Health Statistics and Census population counts. NE = New England; Mid Atl = Mid Atlantic; ENC = East North Central; WNC = West North Central; S Atl = South Atlantic; ESC = East South Central; WSC = West South Central; Mtn = Mountain; Pac = Pacific; App = Appalachia.

Figure 1 shows trends in e_65_ between 1990 and 2016, by metropolitan status (Panel A) and region (Panel B). Table 1 presents values of e_65_ in 2016, as well the measure’s absolute and percent change between 2000 and 2016.

**Table 1.** Life Expectancy at Age 65 (e_65_) in 2016 and Absolute Change Since 2000 by Metro Status, Region, and Sex (% change in parentheses)

	e_65_ in 2016					Absolute change in e_65_ 2000–2016 (% change)				
Sex and region	Large metro	Suburb	Small metro	Nonmetro	*Overall* ^a^	Large metro	Suburb	Small metro	Nonmetro	*Overall* ^a^
**Males**										
Appalachia	17.58	17.71	17.46	16.93	17.30	1.86 (11.8)	1.85 (11.7)	2.13 (13.9)	1.61 (10.5)	1.88 (12.2)
East North Central	17.87	18.39	17.84	17.68	17.95	2.20 (14.0)	2.38 (14.9)	1.93 (12.1)	1.61 (10.0)	2.05 (12.9)
East South Central	17.17	17.30	17.08	16.09	16.81	2.24 (15.0)	2.18 (14.4)	2.05 (13.6)	1.29 (8.7)	1.87 (12.5)
Mid-Atlantic	19.27	19.19	18.45	18.24	19.02	2.74 (16.6)	2.65 (16.0)	2.14 (13.1)	2.29 (14.4)	2.55 (15.5)
Mountain	18.89	19.75	18.97	18.65	18.97	2.09 (12.4)	2.39 (13.8)	1.81 (10.6)	1.76 (10.4)	1.96 (11.5)
New England	18.89	19.10	18.79	18.48	18.86	2.33 (14.1)	2.69 (16.4)	2.32 (14.1)	1.97 (11.9)	2.39 (14.5)
Pacific	20.03	19.20	19.00	18.83	19.51	2.83 (16.4)	2.42 (14.4)	1.95 (11.4)	1.97 (11.7)	2.44 (14.3)
South Atlantic	18.75	19.21	19.02	17.07	18.82	2.53 (15.6)	2.35 (13.9)	2.33 (14.0)	2.09 (14.0)	2.37 (14.4)
West North Central	18.71	18.76	18.39	18.16	18.41	2.68 (16.7)	2.43 (14.9)	1.99 (12.1)	1.60 (9.7)	1.99 (12.1)
West South Central	18.37	18.47	17.64	17.02	17.84	2.40 (15.0)	2.73 (17.4)	1.66 (10.4)	1.48 (9.5)	2.01 (12.7)
Overall^*^	18.96	18.87	18.36	17.54	18.35	2.55 (15.5)	2.43 (14.8)	2.08 (12.8)	1.69 (10.7)	2.29 (14.3)
**Females**										
Appalachia	20.44	20.28	19.99	19.47	19.88	1.60 (8.5)	1.72 (9.3)	1.45 (7.8)	0.87 (4.7)	1.28 (6.9)
East North Central	20.67	20.82	20.51	20.33	20.60	1.97 (10.5)	1.93 (10.2)	1.54 (8.1)	1.13 (5.9)	1.68 (8.9)
East South Central	19.84	19.43	19.71	18.97	19.46	1.81 (10.0)	1.19 (6.5)	1.44 (7.9)	0.66 (3.6)	1.23 (6.7)
Mid-Atlantic	22.35	21.82	21.09	20.93	21.82	2.53 (12.8)	2.41 (12.4)	1.77 (9.2)	1.86 (9.8)	2.30 (11.8)
Mountain	21.62	21.81	21.27	21.15	21.42	2.11 (10.8)	2.20 (11.2)	1.35 (6.8)	1.51 (7.7)	1.72 (8.7)
New England	21.79	21.75	21.56	20.97	21.58	2.01 (10.2)	2.08 (10.6)	2.04 (10.5)	1.55 (8.0)	1.98 (10.1)
Pacific	22.79	21.78	21.56	21.41	22.19	2.71 (13.5)	2.26 (11.6)	1.85 (9.4)	2.06 (10.6)	2.35 (11.8)
South Atlantic	21.93	21.79	21.60	19.94	21.55	2.54 (13.1)	2.19 (11.2)	2.11 (10.8)	1.50 (8.1)	2.17 (11.2)
West North Central	21.28	20.97	20.99	20.79	20.95	1.93 (10.0)	1.77 (9.2)	1.39 (7.1)	0.70 (3.5)	1.24 (6.3)
West South Central	20.96	20.64	20.33	19.58	20.40	2.28 (12.2)	2.25 (12.2)	1.42 (7.5)	0.75 (4.0)	1.63 (8.7)
Overall^*^	21.78	21.37	20.94	20.15	20.94	2.38 (12.3)	2.10 (10.9)	1.71 (8.9)	1.07 (5.6)	1.88 (9.9)

*Note*: Source: Multiple cause of death files from the National Center for Health Statistics and Census population counts.

^a^Overall combines all units in a particular category (e.g., overall value for Appalachia is value for all of Appalachia, regardless of metro status). The value at the intersection of overall and overall is the value for the United States as a whole in the Human Mortality Database.

Among women in both panels of Figure 1, there was little improvement in life expectancy between 1990 and 2000, regardless of region or metro status. The pattern among metro categories for women in Panel A is especially striking. In addition to little or no changes in life expectancy between 1990 and 1999 in any of the four metro categories, there was little differentiation among them. Starting in 1999–2000, however, the period of undifferentiated stasis gave way to a divergence that grew wider over time. Women’s life expectancy in large metro areas began to advance steadily while rates of improvement in nonmetro areas remained modest. The result is that, by 2016, 65-year-old women in large metro areas lived an average of 1.63 years longer than those in nonmetro areas, compared to an advantage of only 0.32 years in 2000 (Table 1). Suburbs did nearly as well as large metro areas, while small metro areas remained roughly half way between the extremes.

The time series for men in Figure 1 are different. Unlike trends in women’s life expectancy, men’s life expectancy by region and metro status improved throughout the entire period. In contrast to women, men in nonmetropolitan areas (Panel A) began with a clear life expectancy deficit in 1990 that slowly became larger throughout the 26-year period. After 2000, large metro areas and their suburbs maintained similar life expectancies while stretching their lead over nonmetro and small metro areas.

Panel B shows that, by 2000, the Pacific region was firmly established as the leader in e_65_ for both sexes, while the East South Central and Appalachian regions ranked at the bottom. Regional patterns of mortality change display a sharp coastal/interior distinction. As shown in Table 1, the four fastest gains in e_65_ between 2000 and 2016 for both sexes occurred in the four coastal regions: Pacific, New England, Mid-Atlantic, and South Atlantic.

In addition to providing values of e_65_ for metro groupings and regions as a whole, Table 1 also includes values for each of the 40 units formed by their combination and for the United States as a whole. The table indicates that e_65_ rose in all units over the recent period, though at different rates: men gained more years than women in 37 out of 40 units, and improvements for both sexes were muted in most nonmetropolitan areas and most of Appalachia and the East South Central region. Communities at the intersection of these categories experienced the smallest gains in life expectancy. Women living in nonmetropolitan areas of the East South Central region, for example, recorded the smallest mortality improvement from 2000 to 2016 of any group in the table, 0.66 years. Nonmetro areas of the East South Central region were also home to men with the smallest improvements, at 1.29 years. Annual values of e_65_ since 1990 for the 10 regions, the 4 metro statuses, the 40 units at their intersection, and the United States as a whole are given in Supplementary Appendix Table 3.

The greatest advance for either sex occurred in large metropolitan areas of the Pacific region. By 2016, 65-year-old men and women in this area outlived their counterparts in the highest mortality area, nonmetro areas in the East South Central region, by nearly 4 years. Adding these years to life expectancy in nonmetropolitan areas in the East South Central regions would have increased life expectancy there by 20% for women and 24% for men. The East South Central region did poorly across the board; it is the only region recording below-average gains in each metro status for both sexes. The only region with greater-than-average life expectancy gains for all metropolitan statuses for both sexes is the Mid-Atlantic region.

**Figure 2.**
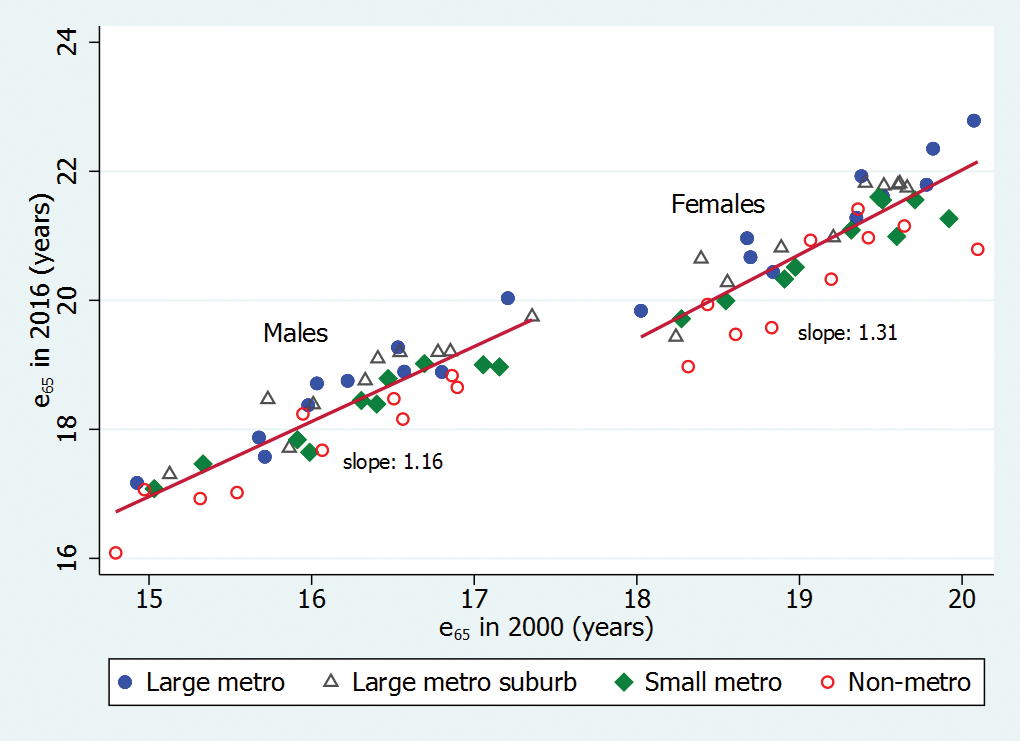
Life expectancy at age 65 (e65) in 2000 and 2016, by sex and metro status. *Note*: Source: Multiple cause of death files from the National Center for Health Statistics and Census population counts. Red lines and slope values are from sex-specific regressions of e65 in 2016 on e65 in 2000 for the 40 units.

Figure 2 shows the relationship between levels of life expectancy in 2000 and 2016 in the 40 areas, with separate regression lines fit to the observations for men and women. Geographic units that began the period with lower life expectancies had, on average, smaller gains in life expectancy over time, especially among women. The slopes of the regression lines, a measure of changes in the degree of inequality in life expectancy, indicate that, for each additional year of e_65_ in 2000, life expectancy over the 16-year period increased by an additional 1.31 years among women and 1.16 years among men. Thus, increases in inequality were greater among women. The figure also distinguishes the four categories of metro areas. Large metropolitan areas are typically located above the regression lines, meaning that they enjoyed greater-than-average increases conditional upon their life expectancy in 2000, while nonmetropolitan areas typically lie below the lines.

### Mortality Change: Metropolitan Status or Region?

We now ask which set of categories, metropolitan status or region, explains more variance in recent changes in e_65_ among all 40 spatial units. Supplementary Appendix Table 4 shows that, for both sexes, mortality change between 2000 and 2016 was more closely associated with metropolitan status than with region. For males, more than half of the variance in mortality change was associated with metropolitan status, about double that of geographic region. These results are surprising because there are only 4 metropolitan statuses to capture variance, versus 10 regions. Regions did, however, dominate the cross section, although their relative importance declined over time.

How are patterns of mortality change affected when metropolitan status and region are considered simultaneously? Table 2 shows the mean predicted changes in e_65_ from linear regressions of mortality change on region and metro status. To compute these values, the predicted changes for one spatial category are averaged across levels of the other spatial variable, keeping the variable of interest constant (the value for large metros, for example, is the average of the predicted change for large metros in all of the 10 regions). This table, therefore, indicates the effects associated with each spatial category, net of the influence of the other spatial category. Comparing these predictions to the actual changes shown in Table 1 indicates that unique spatial pattern persist, even after controlling for region or metro status. The disparities are, however, somewhat reduced in magnitude. For men, the predicted mean difference between large metros and nonmetros when controlling for the fact that large metros and nonmetros are unevenly distributed across regions is 0.62 years, versus 0.86 years in the actual values. For women, the equivalent values are 0.89 versus 1.31 years. Some of the disadvantage of the Appalachian region, for example, is a result of its high proportion of residents in nonmetropolitan areas, while some of the advantage of those in large metropolitan areas is a result of their disproportionate location on the coasts.

**Table 2.** Mean Predicted Values of Change in Life Expectancy at Age 65 (e_65_) Between 2000 and 2016 by Sex, by Metro Status and Region

	Males		Females	
Spatial unit	Mean predicted change	95% CI	Mean predicted change	95% CI
**Metro status**				
Large metro	2.39	2.24–2.53	2.15	2.00–2.30
Suburbs	2.41	2.26–2.55	2.00	1.85–2.15
Small metro	2.03	1.88–2.17	1.64	1.48–1.79
Nonmetro	1.77	1.62–1.91	1.26	1.10–1.41
**Region**				
Appalachia	1.86	1.63–2.09	1.41	1.17–1.65
East North Central	2.03	1.80–2.26	1.64	1.40–1.88
East South Central	1.94	1.71–2.17	1.28	1.03–1.52
Mid-Atlantic	2.46	2.23–2.69	2.14	1.90–2.39
Mountain	2.01	1.78–2.24	1.79	1.55–2.03
New England	2.33	2.10–2.55	1.92	1.68–2.16
Pacific	2.29	2.06–2.52	2.22	1.98–2.46
South Atlantic	2.33	2.10–2.55	2.09	1.85–2.33
West North Central	2.17	1.94–2.40	1.45	1.21–1.69
West South Central	2.07	1.84–2.30	1.68	1.44–1.92

*Note*: Source: Multiple cause of death files from the National Center for Health Statistics and Census population counts.

Estimated from linear regressions of change in e_65_ on metro and region status (40 units). Predicted changes for one spatial category averaged across levels of the other spatial variable, keeping variable of interest constant (value for large metros, for example, is average of predicted change for large metros in all 10 regions) (*margins* package in Stata).

CI = confidence interval.

### Causes of Death and Changes in e_65_

We now turn to an analysis of the causes of death responsible for improvements in e_65_ as well as those creating spatial divergence. Panel A of Table 3 shows the absolute contribution of 10 causes of death to changes in e_65_ between 2000 and 2016 by metropolitan status and sex (the percent contribution to overall changes is given in parentheses). For both sexes, the net gains in e_65_ in all four metropolitan categories documented above were primarily a result of significant improvements in circulatory disease mortality. Among men in all metro types, declines in mortality from circulatory diseases represented at least 2/3 of the 1.7–2.6 year gain in e_65_. Among women, circulatory diseases contributed 83%–140% of the smaller 1.1–2.4 year gains.

**Table 3.** Absolute Cause-Specific Contributions to Changes in Life Expectancy at Age 65 (e_65_) Between 2000 and 2016 by Sex, by Metro Status, and Region (% contributions in parentheses)

		Absolute contribution by cause of death (% contribution in parentheses)									
Spatial unit and sex	Change in e_65_, 2000– 2016	Smoking- related	Screenable cancers	Other cancers	Mental/ nervous	Alzheimer’s disease	Circulatory diseases	Influenza/ pneumonia	Diabetes	External causes	All other causes
**Panel A: Metro Status**											
**Males**											
LCM	2.55	0.50 (19.8)	0.25 (9.7)	0.16 (6.5)	−0.23 (−9.1)	−0.10 (−4.1)	1.72 (67.4)	0.15 (5.9)	0.03 (1.0)	−0.04 (−1.4)	0.11 (4.3)
LMS	2.43	0.52 (21.4)	0.24 (9.9)	0.16 (6.4)	−0.25 (−10.4)	−0.06 (−2.4)	1.62 (66.6)	0.13 (5.4)	0.05 (2.1)	−0.03 (−1.2)	0.05 (2.2)
Small Metro	2.08	0.46 (22.3)	0.23 (11.1)	0.11 (5.4)	−0.25 (−12.2)	−0.09 (−4.5)	1.49 (71.7)	0.12 (5.7)	0.04 (1.8)	−0.04 (−1.9)	0.01 (0.5)
Nonmetro	1.69	0.35 (20.6)	0.20 (11.7)	0.09 (5.5)	−0.24 (−13.9)	−0.09 (−5.1)	1.33 (79.0)	0.11 (6.4)	0.00 (0.1)	−0.03 (−1.9)	−0.04 (−2.3)
***LCM—Nonmetro***	0.86	0.15	0.05	0.07	0.01	−0.01	0.39	0.04	0.03	−0.01	0.15
**Females**											
LCM	2.38	0.26 (11.0)	0.18 (7.6)	0.16 (6.9)	−0.38 (−16.0)	−0.26 (−11.1)	1.98 (83.4)	0.18 (7.4)	0.10 (4.4)	−0.04 (−1.5)	0.19 (7.8)
LMS	2.10	0.18 (8.7)	0.19 (9.0)	0.17 (7.9)	−0.44 (−21.0)	−0.18 (−8.4)	1.81 (86.4)	0.14 (6.9)	0.10 (4.9)	−0.03 (−1.6)	0.15 (7.1)
Small Metro	1.71	0.11 (6.4)	0.16 (9.1)	0.16 (9.1)	−0.42 (−24.7)	−0.26 (−15.0)	1.68 (98.4)	0.14 (8.3)	0.10 (6.0)	−0.04 (−2.5)	0.09 (5.0)
Nonmetro	1.07	−0.11 (−10.6)	0.14 (12.9)	0.10 (9.6)	−0.44 (−41.0)	−0.26 (−24.1)	1.50 (140.0)	0.12 (11.2)	0.08 (7.3)	−0.04 (−3.4)	−0.02 (−2.0)
***LCM—Nonmetro***	1.31	0.37	0.04	0.06	0.06	0.00	0.48	0.06	0.02	0.00	0.21
**Panel B: Region**											
**Males**											
Appalachia	1.88	0.40 (21.2)	0.20 (10.7)	0.10 (5.3)	−0.25 (−13.5)	−0.09 (−4.7)	1.45 (77.2)	0.08 (4.4)	0.03 (1.8)	−0.03 (−1.8)	−0.01 (−0.6)
E. N. Central	2.05	0.37 (18.0)	0.24 (11.7)	0.14 (6.7)	−0.24 (−11.7)	−0.07 (−3.5)	1.47 (71.5)	0.10 (5.0)	0.04 (1.9)	−0.03 (−1.6)	0.04 (1.8)
E. S. Central	1.87	0.42 (22.3)	0.25 (13.2)	0.10 (5.2)	−0.23 (−12.6)	−0.11 (−5.9)	1.38 (74.1)	0.10 (5.3)	0.00 (0.0)	0.00 (−0.1)	−0.03 (−1.5)
Mid−Atlantic	2.55	0.45 (17.5)	0.29 (11.3)	0.14 (5.5)	−0.27 (−10.6)	−0.03 (−1.3)	1.74 (68.3)	0.13 (4.9)	0.06 (2.2)	−0.02 (−1.0)	0.08 (3.1)
Mountain	1.96	0.49 (25.1)	0.21 (10.9)	0.10 (5.0)	−0.17 (−8.8)	−0.09 (−4.5)	1.24 (63.2)	0.16 (8.0)	0.00 (0.0)	−0.04 (−2.0)	0.06 (3.1)
New England	2.39	0.54 (22.5)	0.28 (11.7)	0.19 (7.8)	−0.34 (−14.4)	−0.01 (−0.3)	1.56 (65.1)	0.15 (6.4)	0.04 (1.8)	−0.07 (−2.7)	0.05 (2.3)
Pacific	2.44	0.56 (23.1)	0.19 (7.8)	0.12 (5.0)	−0.21 (−8.8)	−0.15 (−6.0)	1.75 (71.9)	0.18 (7.5)	0.00 (0.0)	−0.03 (−1.3)	0.02 (0.8)
S. Atlantic	2.37	0.51 (21.6)	0.24 (10.3)	0.14 (6.0)	−0.26 (−10.9)	−0.08 (−3.5)	1.63 (68.8)	0.12 (5.1)	0.03 (1.2)	−0.03 (−1.3)	0.06 (2.7)
W. N. Central	1.99	0.38 (19.1)	0.22 (11.3)	0.12 (5.9)	−0.25 (−12.8)	−0.06 (−3.0)	1.45 (73.1)	0.13 (6.7)	0.02 (1.1)	−0.04 (−2.0)	0.01 (0.4)
W. S. Central	1.88	0.48 (23.8)	0.19 (9.5)	0.13 (6.3)	−0.23 (−11.6)	−0.13 (−6.3)	1.40 (69.6)	0.12 (6.1)	0.07 (3.3)	−0.02 (−1.2)	0.01 (0.5)
***Slope***^a^	--	0.16	0.07	0.07	−0.08	0.05	0.56	0.06	0.02	−0.02	0.09
**Females**											
Appalachia	1.28	−0.07 (−5.6)	0.15 (11.5)	0.15 (11.6)	−0.45 (−35.5)	−0.27 (−21.2)	1.59 (124.8)	0.11 (8.4)	0.10 (8.1)	−0.04 (−2.7)	0.01 (0.6)
E. N. Central	1.68	0.04 (2.6)	0.18 (10.6)	0.13 (8.0)	−0.43 (−25.3)	−0.21 (−12.8)	1.70 (101.3)	0.11 (6.8)	0.12 (7.1)	−0.04 (−2.3)	0.07 (3.9)
E. S. Central	1.23	−0.09 (−7.5)	0.12 (9.9)	0.08 (6.7)	−0.41 (−33.4)	−0.36 (−28.9)	1.67 (135.9)	0.13 (10.4)	0.07 (6.0)	−0.01 (−1.0)	0.03 (2.0)
Mid-Atlantic	2.30	0.13 (5.7)	0.22 (9.5)	0.17 (7.3)	−0.48 (−20.7)	−0.10 (−4.6)	1.99 (86.7)	0.13 (5.8)	0.10 (4.3)	−0.01 (−0.5)	0.15 (6.4)
Mountain	1.72	0.15 (9.0)	0.14 (8.3)	0.16 (9.1)	−0.24 (−13.8)	−0.24 (−13.9)	1.37 (80.0)	0.15 (8.6)	0.08 (4.4)	−0.06 (−3.4)	0.20 (11.7)
New England	1.98	0.29 (14.7)	0.24 (12.2)	0.20 (9.9)	−0.68 (−34.4)	−0.05 (−2.7)	1.65 (83.4)	0.20 (10.0)	0.09 (4.7)	−0.06 (−2.9)	0.10 (5.1)
Pacific	2.35	0.35 (14.8)	0.15 (6.5)	0.15 (6.4)	−0.31 (−13.1)	−0.37 (−15.9)	2.02 (86.2)	0.23 (9.9)	0.05 (2.1)	−0.04 (−1.9)	0.12 (4.9)
S. Atlantic	2.17	0.21 (9.9)	0.16 (7.4)	0.14 (6.5)	−0.43 (−19.8)	−0.19 (−8.8)	1.88 (86.4)	0.14 (6.5)	0.10 (4.6)	−0.04 (−1.9)	0.20 (9.3)
W. N. Central	1.24	−0.08 (−6.2)	0.15 (12.0)	0.12 (9.7)	−0.48 (−38.3)	−0.21 (−17.3)	1.57 (126.2)	0.15 (11.7)	0.08 (6.4)	−0.07 (−5.7)	0.02 (1.5)
W. S. Central	1.63	0.09 (5.2)	0.13 (7.8)	0.16 (9.5)	−0.39 (−23.6)	−0.35 (−21.7)	1.62 (99.0)	0.15 (9.1)	0.16 (9.8)	−0.01 (−0.8)	0.09 (5.5)
*** Slope*** ^a^	--	0.32	0.05	0.04	0.00	0.08	0.34	0.05	−0.01	0.01	0.12

*Note*. LCM = Large central metro; LMS = Large metro suburb. Source: Multiple cause of death files from the National Center for Health Statistics and Census population counts.

Smoking-related: lung cancer and respiratory diseases. Screenable cancers: Breast, prostate, cervical, and colorectal cancers.

^a^Slope: To identify causes of death responsible for changing regional differentials in life expectancy, we regress the contribution of each cause for a region on the total change in life expectancy for that region. The slope of each regression indicates the contribution of a particular cause to changes in all-cause mortality differentials. Added across causes, the slopes sum to 1.00 (Preston, 1976).

Improvement in smoking-related mortality (lung cancer and respiratory diseases) was the second largest contributor to life expectancy gains among men across metro categories, with 65-year-old men gaining between 0.35 and 0.50 additional years of life from reductions in mortality from this cause of death. Smoking-related mortality also made the second greatest contribution among women in large central metros, but its contribution was substantially lower in other metropolitan statuses.

Two causes of death thought to be strongly influenced by the quality of medical services, screenable cancers and influenza/pneumonia, helped advance life expectancy in all metropolitan categories by 0.11–0.25 years. On the other hand, rising mortality from Alzheimer’s disease and other diseases of the mental or nervous systems, which include many non-Alzheimer’s forms of dementia, were negative contributors to changes in life expectancy. The setbacks were especially prominent among women, ranging from −0.18 years to −0.44 years.

In addition to accounting for most of the improvements in mortality over the period, circulatory diseases were also responsible for a significant portion of changes in the gap between large central metros and nonmetro areas. Panel A of Table 3 shows the differences in cause-specific contributions between large and nonmetro areas. Men in large metros gained 0.39 more years from improvements in circulatory disease mortality than did men in nonmetro areas, a substantial portion of the 0.86 year gap in their overall gains. Women in large metro areas gained 1.31 more years of life than women in nonmetro areas, 0.48 years of which were due to more substantial improvements in circulatory disease mortality in the former. Women in large metros also gained 0.37 more years from changes in smoking-related mortality than their counterparts in nonmetro areas, for whom smoking-related mortality actually *rose*. In contrast, men in all metropolitan statuses enjoyed substantial gains in life expectancy from declines in this cause of death, with a difference of only 0.17 years across the metropolitan spectrum. Between them, differential gains in circulatory disease and smoking-related mortality account for 65% of the growing gap in life expectancy gains among men and 63% among women.

Sex differences in life expectancy narrowed during the period in all metro categories, but the changes were highly differentiated. As shown in Table 1, men gained 0.17 years on women in large metropolitan areas and more than three times as much in nonmetro areas at 0.62 years; suburbs and small metros were in between. In all four metropolitan categories, differential improvements in smoking-related mortality by sex accounted for at least 3/4 of declining sex differences in life expectancy.

Panel B of Table 3 presents information equivalent to Panel A but for geographic regions. As in metro groups, improvements in mortality from circulatory diseases accounted for the majority of improvements in life expectancy in all regions. The two regions with the largest gains attributable to circulatory diseases, the Mid-Atlantic and Pacific regions, also had the largest gains in e_65_ overall. The three smallest gains in e_65_ occurred in Appalachia, East South Central, and West North Central. For women in these regions, years added due to declining circulatory disease mortality actually exceeded the total improvement in life expectancy, in part because a growing death rate from smoking-related deaths cost women months of life in these (and only these) regions. Unusually small gains from screenable cancers and influenza/pneumonia in Appalachia added to its disadvantage and suggest a possible role for medical services.

All regions suffered substantial reductions in life expectancy by virtue of rising mortality from disorders of the mental or nervous systems. That rise was largest in New England for both sexes. At the same time, the contribution of rising Alzheimer’s mortality was smallest in New England. This relationship suggests that diagnosis and coding practices for dementia deaths in New England may have changed in such a way that they are less likely to be assigned to Alzheimer’s there than elsewhere. Supporting this inference, Massachusetts had the lowest percentage of dementia deaths assigned to Alzheimer’s in 2017 and Connecticut the third lowest of any state. The two states had the highest percentage assigned to “unspecified dementia,” a component of our “disorders of the mental or nervous systems” category (Kramarow & Tejada-Vera, 2019).

The slope of the cause-specific regressions of each region’s cause contribution on change in e_65_ is also shown in Panel B. The slope can be interpreted as the proportion of the regional variation in changes in e_65_ that is associated with a particular cause. For both sexes, differential improvements in death rates from circulatory diseases and smoking-related deaths were the largest contributors to regional differences in life expectancy change, together accounting for at least 2/3 of regional disparities. Circulatory diseases were responsible for more than half of the differences among men, whereas these causes drove about a third of the variation for women. Screenable cancers and influenza/pneumonia, two categories believed to reflect the quality of medical services, contributed 13% of the changing regional differentials for men and 10% for women.

Circulatory diseases and diseases associated with smoking were the principal drivers of spatial differentiation in life expectancy during the period for both metropolitan categories and geographic regions. That result is maintained if the slope values are calculated based on cause decompositions in all 40 units, rather than just the 4 metro types or 10 regions. Together, differential improvements in circulatory disease and smoking-related mortality across the 40 units accounted for 64% of spatial disparities in life expectancy change among women (34% and 30%, respectively) and 67% among men (47% and 20%; results not shown).

## Discussion

In contrast to widespread mortality increases during the working ages in the past decade or more (Case & Deaton, 2015, 2017; Elo et al., 2019; Shiels et al., 2017; Woolf et al., 2018), trends in life expectancy at age 65 have been favorable. Between 2000 and 2016, the measure increased for both sexes in each of the 40 spatial units that we investigate. However, increases were not uniform across space. Nonmetropolitan areas had smaller gains than large metropolitan areas, and coastal areas gained more years of life than the interior. In general, areas that began the period with favorable mortality conditions at older ages experienced larger mortality improvements between 2000 and 2016.

Reductions in mortality from circulatory diseases accounted for a majority of the gain in life expectancy at age 65 for men and women in all metropolitan statuses and regions (Table 3). This cause of death was also primarily responsible for the spatial divergence in mortality trends during the period, although differential improvements in deaths related to smoking were nearly as important for women. Smoking-related mortality actually rose among women in nonmetropolitan areas, Appalachia, and the East South Central and West North Central regions, while falling in all other regions and metropolitan statuses. Men narrowed women’s lead in e_65_ in all metropolitan categories; in all cases, greater reductions in smoking-related diseases among men accounted for at least 3/4 of the relative male improvement.

The importance of smoking-related diseases in patterning spatial change in mortality at older ages reflects the historically high prevalence of smoking in the United States, the associated mortality risk, and the decades-long lag between smoking behavior and disease onset (de Groot, Wu, Carter, & Munden, 2018; Oza et al., 2011). The evolution of geographic smoking patterns over time is also consistent with the patterns revealed. In the first national probability survey of U.S. smoking behavior, taken in 1955, women in urbanized environments were more likely than women in rural farm areas to be current smokers (28% vs 9%), as were women who lived in the West and Northeast (relative to those in the North Central and South) (Haenszel, Shimkin, & Miller, 1956: Table 14b). These metropolitan and regional patterns reversed with time. By the mid-2010’s, rural women and those living in the Midwest and South were most likely to be smokers (Jamal et al., 2015; Roberts et al., 2017). The much larger reductions in smoking prevalence in metropolitan areas and coastal populations are consistent with more rapid improvements in mortality from lung cancer/respiratory diseases among women in these areas. Among men, regional differentiation in smoking prevalence is and has been muted (Haenszel et al., 1956; Roberts et al., 2017).

Smoking is also a major risk factor for circulatory disease (Oza et al., 2011). Some of the reduction in circulatory disease mortality that we have observed is undoubtedly attributable to reductions in smoking prevalence. Other factors at work include improvements in medical prevention and treatment of high blood pressure and serum cholesterol, along with surgical advances (Chernew, Cutler, Ghosh, & Landrum, 2016; Di Cesare et al., 2013; Ford et al., 2007; Stewart & Cutler, 2014). Large metropolitan areas, along with the Pacific and Mid-Atlantic regions, saw larger life expectancy improvements from circulatory diseases than other spatial units. These areas may have benefitted disproportionately from medical and surgical advances, a pattern consistent with declining numbers of physicians and hospitals in rural areas in the past decade (Cecil G. Sheps Center, 2019; Orlowski & Dill, 2017). The fact that two categories thought to be unusually responsive to the quality of medical services, screenable cancers and influenza/pneumonia, contributed 9%–13% to the widening metropolitan and regional disparities provides additional evidence of the possible role of medical services. The longevity successes of large coastal metropolitan areas may be part of a broader social transformation that reflects the increasing value of density in a knowledge economy, a value embedded in “superstar cities” (Le Galès & Pierson, 2019).

Changes in diagnostic and coding practices have likely affected some of the trends that we have identified in cause-specific mortality. Perhaps the most serious issue pertains to dementia. Coding changes implemented by the National Center for Health Statistics in 2006 produced an increase in deaths from “unspecified dementia” and “vascular dementia,” components of our category “disorders of the mental or nervous systems.” This increase occurred at the expense of anemias, nutritional deficiencies, heart disease, and cerebrovascular disease (Kramarow & Tejada-Vera, 2019).

To place U.S. patterns in a broader context, Supplementary Appendix Table 5 presents levels of and change in life expectancy at age 65 for the United States and comparison countries, using data from the Human Mortality Database (Human Mortality Database, 2019). We include the 16 Organization of Economic Cooperation and Development (OECD) member countries with a population size of at least five million in 2010 and available data on e_65_ in 2016. To highlight variation within the United States, the Table also includes the two U.S. spatial units with the highest and lowest values of e_65_ in 2016 (large metros in the Pacific and nonmetro areas in East South Central).

Compared to other OECD countries, the United States had both low levels of e65 in 2000 and unusually slow gains therein between 2000 and 2016. In 2016, e_65_ among U.S. men ranked 9th out of 17 countries (excluding the two U.S. spatial units); women ranked 12th, lagging 3.5 years behind the leader Japan. While the deteriorating position of the United States is a continuation of a process of unusually slow improvements in mortality at older ages dating back to the 1980’s (National Research Council, 2011; Palloni & Yonker, 2016), this paper suggests that a new element of increased spatial dispersion was added around the turn of the 21st century. Indeed, the poor ranking of the United States as whole is largely attributable to the poor performance of nonmetropolitan and small metropolitan areas more broadly. If large metro areas in the Pacific region are ranked as their own country, men in this area rank first and women fourth. At the other end of the spectrum, men in nonmetro areas in the East South Central region tie with the Czech Republic and outrank only Poland and Hungary. Women in this unit rank second-last, ahead of only Hungary. Gains in life expectancy for both men and women living in nonmetro areas more broadly (Table 1) were lower than those in any OECD comparison country.

This analysis identified spatial variation in levels and changes in mortality at older ages. A logical follow-up question asks which proximate factors drive the observed patterns. Although the results by cause of death offer important insight into the intermediate stage between mortality and its “ultimate causes,” this analysis does not directly explore the many possible upstream factors. Virtually all of the scores of variables known to affect mortality are also active on the spatial landscape. Obesity levels, for example, rose throughout the period and today are highest in Appalachia (Behavioral Risk Factor Surveillance System, 2019). Nevertheless, mortality from diabetes, for which obesity is a major risk factor, left little mark on changes in mortality at older ages in this region or elsewhere (Table 3). Other factors that are undoubtedly operating on spatial patterns during the period are compositional changes in educational attainment, personal income, insurance coverage, and race. Blacks face higher mortality rates than whites, but have experienced faster gains in life expectancy since 2000 (Arias and Xu, 2018). The largest concentration of blacks as a proportion of the population is in the East South Central division (Rastogi, Johnson, Hoeffel, & Drewery, 2011). While this concentration may help to account for high mortality in this region, it cannot account for the region’s relatively slow improvement. Furthermore, it is not race itself that shapes mortality risks, but rather the composite of many other variables for which race is a proxy.

This is not the first study to examine changing geographic patterns of mortality in the United States. Several other studies have used county-level data to examine trends in life expectancy at birth or age-standardized death rates for all ages combined (Cosby et al., 2008; Dwyer-Lindgren et al., 2016; Ezzati, Friedman, Kulkarni, & Murray, 2008; Singh and Siahpush, 2014; Wang, Schumacher, Levitz, Mokdad, & Murray, 2013). However, this is the first study to focus on geographic trends in mortality at older ages. While geographic inequalities in mortality are highest at younger adult ages (Vierboom, Preston, & Hendi, 2019), we find that important inequalities in the length of life remain at older ages. These inequalities have grown wider over time, even as mortality levels themselves have declined. The growing number of Americans who are reaching age 65 face a future of widening inequalities.

## Supplementary Material

gbz160_suppl_Supplementary_Appendix

gbz160_suppl_Appendix_Tables
