# Supplementary material for: Life Beyond 65: Changing Spatial Patterns of Survival at Older Ages in the United States, 2000–2016
Source: J Gerontol B Psychol Sci Soc Sci. 2020 Jan 29;75(5):1093–103. doi: 10.1093/geronb/gbz160 (PMC7161363; doi:10.1093/geronb/gbz160)
Supplement: gbz160_suppl_Appendix_Tables [file gbz160_suppl_appendix_tables.docx]

**Appendix Table 1.** Definitions of region and metropolitan status.

|  |  |
| --- | --- |
| **Region** | New England (CT, ME, MA, NH, RI, VT) |
|  | Mid-Atlantic (NJ, NY, PA) |
|  | East North Central (IL, IN, MI, OH, WI) |
|  | West North Central (IA, KS, MN, MO, NE, ND, SD) |
|  | South Atlantic (DE, DC, FL, GA, MD, NC, SC, VA) |
|  | East South Central (AL, KY, MS, TN) |
|  | West South Central (AR, LA, OK, TX) |
|  | Mountain (AZ, CO, ID, MT, NV, NM, UT, WY) |
|  | Pacific (AK, CA, HW, OR, WA) |
|  | Appalachia^a^ |
|  |  |
|  |  |
| **Metro Status**^b^ | Large central metro (counties of MSAs with a population of at least 1 million, including counties that contain all or a part of the area’s inner cities) |
|  | Large metro suburb (surrounding counties of large central metro) |
|  | Medium & small metro (counties with MSAs of 50,000-999,999 population) |
|  | Non-metropolitan areas |
|  |  |

*Note.* MSA: Metropolitan Statistical Area.

a. As defined by the Appalachian Regional Commission and used by Elo et al. (2019) and Vierboom, Preston, & Hendi (2019). Appalachia includes all of WV and certain counties in AL, GA, KY, MD, MS, NY, NC, OH, PA, SC, TN, and VA. These counties are excluded from their overlapping census divisions.

b. Based on the NCHS metropolitan classification scheme (Ingram & Franco, 2014).

**Appendix Table 2.** Cause of death classifications.

| **Cause of death** | **ICD-10 codes** |
| --- | --- |
| Alzheimer’s disease | G30 |
| Breast, prostrate, cervical, and colorectal cancers (“screenable cancers”) | C50, C53, C61, C18-C21 |
| Circulatory diseases | I00–I99 (excl. I42.6) |
| Diabetes | E10-E14 (excl. E24.4) |
| External causes (incl. substance use disorders) | E24.4, F10, G31.2, G62.1, G72.1, I42.6, K29.2, K70, K85.2, K86.0, R78.0, V01-Y89 |
| Influenza and pneumonia | J09-J18 |
| Lung cancer and respiratory diseases (excl. influenza and pneumonia) (“smoking-related conditions”) | C33-C34, J00–J98 (excl. J09–J18) |
| Mental and nervous system disorders (excl. Alzheimer’s Disease and substance use disorders) | F01–F99 (excluding F10), G00–G98 (excluding G30, G31.2, G62.1, G72.1) |
| Other cancers | C00-D48 (excl. C33-C34, C50, C53, C61, C18-21) |
| All other causes | A00-B99, D49-E90 (excl. E10-E14), G99, H00-H93, J99-U99 (excl. K29.2, K70, K85.2, K86.0, R78.0), Y90-Y98 |

**Appendix Table 3.** *See attached Excel file.*

**Appendix Table 4**. Percentage of variation in life expectancy at age 65 (e_65_) levels and change among 40 units associated with region and metro status.

| **Sex and spatial unit** | **Change in e_65_ 2000-2016** (%) | **e_65_ in  2000** (%) | **e_65_ in  2016** (%) |
| --- | --- | --- | --- |
|  |  |  |  |
| **Males** |  |  |  |
| Region | 24.7 | 80.9 | 70.4 |
| Metro | 51.1 | 4.0 | 19.8 |
|  |  |  |  |
|  |  |  |  |
| **Females** |  |  |  |
| Region | 38.2 | 79.4 | 72.7 |
| Metro | 47.1 | 1.2 | 18.9 |
|  |  |  |  |

*Note.* Source: Multiple cause of death files from the National Center for Health Statistics and Census population counts.

Percentages = (R^2^ value * 100) from bivariate linear regressions of life expectancy measure on region or metro status.

**Appendix Table 5**. Life expectancy at age 65 (e_65_) in 2016 and absolute change since 2000 for 17 countries and the best- and worst-performing US spatial units, with rankings.

| **Sex and country** | **e_65_ in 2016** | **Change in e_65_ 2000-2016** | **Ranking…** | | |  |
| --- | --- | --- | --- | --- | --- | --- |
|  |  |  | of level in 2000 | of level in 2016 | of change 2000-2016 |  |
| **Males** |  |  |  |  |  |  |
| *Pacific large central metros* | 20.03 | 2.83 | 2 | 1 | 5 |  |
| Australia | 19.85 | 2.80 | 3 | 2 | 6/7 |  |
| Switzerland | 19.75 | 2.80 | 4 | 3 | 6/7 |  |
| Japan | 19.59 | 2.09 | 1 | 4 | 17 |  |
| Canada | 19.56 | 2.87 | 6/7 | 5 | 4 |  |
| Israel | 19.46 | 2.57 | 5 | 6 | 9 |  |
| France | 19.32 | 2.66 | 8 | 7 | 8 |  |
| Spain | 19.10 | 2.45 | 9 | 8 | 11 |  |
| Sweden | 19.01 | 2.32 | 6/7 | 9 | 14 |  |
| UK | 18.60 | 2.95 | 12 | 10 | 2 |  |
| Netherlands | 18.37 | 3.05 | 14 | 11 | 1 |  |
| U.S.A. | 18.35 | 2.29 | 10 | 12 | 16 |  |
| Austria | 18.29 | 2.33 | 11 | 13 | 13 |  |
| Denmark | 18.11 | 2.94 | 15 | 14 | 3 |  |
| Germany | 17.88 | 2.31 | 13 | 15 | 15 |  |
| Czech Republic | 16.09 | 2.47 | 16/17 | 16/17 | 10 |  |
| *East South Central non-metros* | 16.09 | 1.29 | 16/17 | 16/17 | 19 |  |
| Poland | 15.86 | 2.42 | 18 | 18 | 12 |  |
| Hungary | 14.58 | 1.70 | 19 | 19 | 18 |  |
|  |  |  |  |  |  |  |
|  |  |  |  |  |  |  |
| **Females** |  |  |  |  |  |  |
| Japan | 24.43 | 2.08 | 1 | 1 | 9 |  |
| France | 23.19 | 1.97 | 2 | 2 | 12 |  |
| Spain | 23.00 | 2.31 | 4 | 3 | 6 |  |
| *Pacific large central metros* | 22.79 | 2.71 | 7 | 4 | 2 |  |
| Switzerland | 22.54 | 1.84 | 3 | 5 | 15 |  |
| Australia | 22.53 | 1.95 | 5 | 6 | 13 |  |
| Canada | 22.37 | 2.09 | 6 | 7 | 7/8 |  |
| Israel | 21.65 | 2.55 | 12 | 8 | 4 |  |
| Austria | 21.52 | 2.04 | 9 | 9 | 10 |  |
| Sweden | 21.47 | 1.40 | 8 | 10 | 18 |  |
| Germany | 21.11 | 1.73 | 10 | 11 | 17 |  |
| Netherlands | 21.01 | 1.80 | 11 | 12 | 16 |  |
| UK | 20.97 | 2.09 | 14 | 13 | 7/8 |  |
| U.S.A. | 20.94 | 1.88 | 13 | 14 | 14 |  |
| Denmark | 20.73 | 2.51 | 16 | 15 | 5 |  |
| Poland | 20.13 | 2.73 | 17 | 16 | 1 |  |
| Czech Republic | 19.75 | 2.66 | 18 | 17 | 3 |  |
| *East South Central non-metros* | 18.97 | 0.66 | 15 | 18 | 19 |  |
| Hungary | 18.58 | 1.99 | 19 | 19 | 11 |  |
|  |  |  |  |  |  |  |

*Note*. Source: Human Mortality Database for national numbers. Multiple cause of death files from the National Center for Health Statistics and Census population counts for spatial units.

Large central metros in the Pacific had the highest e_65_ in 2016 and non-metro areas in the East South Central region the lowest.
